# The relationship of serum alanine aminotransferase normal-range levels to arterial stiffness and metabolic syndrome in non-drinkers and drinkers: a Chinese community-based analysis

**DOI:** 10.1186/s12876-017-0607-8

**Published:** 2017-04-11

**Authors:** Shihui Fu, Ying Lin, Leiming Luo, Ping Ye

**Affiliations:** 1 Department of Geriatric Cardiology, Chinese People’s Liberation Army General Hospital, Beijing, 100853 China; 2 Department of Cardiology and Hainan Branch, Chinese People’s Liberation Army General Hospital, Beijing, China

**Keywords:** Alanine aminotransferase, Alcohol intake, Arterial stiffness, Chinese community-based analysis, Metabolic syndrome

## Abstract

**Background:**

Few studies have investigated the relationship between carotid-femoral pulse wave velocity (cfPWV) and serum alanine aminotransferase (ALT) normal-range levels across the world. The current analysis was designed to explore the relationship of serum ALT normal-range levels to cfPWV and metabolic syndrome (Mets) in non-drinkers and drinkers in a Chinese community-dwelling population.

**Methods:**

There were 2202 participants with serum ALT levels within normal range enrolled for the current analysis.

**Results:**

Median (range) age of participants was 53 (18–96) years, 51.5% were males, and 31.2% were drinkers. Prevalence of Mets was 29.4%. Median (range) of cfPWV was 10.1 (2.4-32.7) m/s. Hazard ratios for prevalence of Mets, central obesity and high triglyceride significantly increased with elevated levels of serum ALT in both non-drinkers and drinkers (*p* < 0.05 for all). Hazard ratios for prevalence of cfPWV > 10 m/s, high blood pressure and high blood glucose significantly increased with elevated levels of serum ALT in non-drinkers (*p* < 0.05 for all), but not in drinkers (*p* ≥ 0.05 for all).

**Conclusions:**

In a Chinese community-dwelling population, prevalence of Mets and its components (including central obesity and high TG) increased with an elevation in serum ALT levels within normal range in both non-drinkers and drinkers, while cfPWV and other components of Mets, such as high blood pressure and glucose, increased with an elevation in serum ALT levels in non-drinkers, but not in drinkers.

## Background

Prevalence of Metabolic syndrome (Mets) is rapidly increasing in parallel with an elevation in serum levels of alanine aminotransferase (ALT) [1]. Globally, measurements of serum ALT levels are the most commonly used test to identify patients suffering from liver disease. Serum ALT levels also act as an important marker for liver function and disease severity [2]. In previous studies, Mets has been shown to be related to serum ALT levels [3–6]. However, published studies generally include clinical patients with elevated levels of serum ALT, and there is little information on the relationship between Mets and serum ALT levels within normal range in Chinese community-dwelling population [7].

Carotid-femoral pulse wave velocity (cfPWV) is a widely accepted measure of central arterial stiffness, which is commonly used as a marker of atherosclerosis [2]. However, to our knowledge, few studies have investigated the relationship between cfPWV and serum ALT levels within normal range across the world. Moreover, alcohol intake has an important effect on the relationship of serum ALT levels to cfPWV and Mets. Therefore, the current analysis was designed to explore the relationship of serum ALT levels within normal range to cfPWV, Mets and its components in non-drinkers and drinkers in a Chinese community-dwelling population.

## Methods

### Study population

**Fig. 1:**
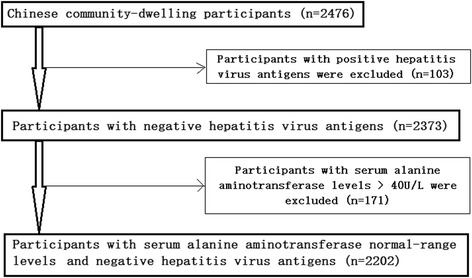
Flow chart of study participant selection process

A stratified cluster sampling method was used in the selection of participants through a routine health check-up in Beijing between May 2007 and July 2009. In the first layer of sampling, three out of 18 Beijing districts were selected (Fengtai, Shijingshan and Daxing); in the second layer of sampling, four communities were selected from the three selected districts; in the third layer of sampling, participants aged 18 years or over were selected from the four selected communities. The current analysis was conducted in 2476 Chinese community-dwelling participants. After the exclusion of 103 participants with positive hepatitis virus antigens, there were 2373 participants with negative hepatitis virus antigens. After the exclusion of 171 participants with serum ALT levels > 40 U/L, other 2202 participants were enrolled for the final analysis (Fig. 1).

### Participant interview and serological test

Participants were interviewed by trained physicians at the local health service center. Systolic and diastolic blood pressure (SBP and DBP) were measured twice for each participant in the sitting position, with an interval of at least 1 min between measurements, using a standard sphygmomanometer (Yuwell medical equipment & supply Co., Ltd., Jiangsu, China). A cuff of appropriate size was placed on the right arm of participants, after a rest period for at least 5 min. In order to assess central obesity, waist circumference (WC) was measured from the standing participants midway between the lowest rib and iliac crest using a soft tape. Blood samples were taken from participants for serological tests. Overnight fasting serum samples were tested by qualified personnel in our central laboratory. Fasting blood glucose (FBG), triglyceride (TG), high-density lipoprotein-cholesterol (HDL-c), ALT and hepatitis virus antigens were assessed using the assays (Roche Products Ltd, Basel, Switzerland) on a full automatic biochemical autoanalyser (COBAS 6000; Roche Products Ltd, Basel, Switzerland).

### Diagnostic criteria

Central obesity was defined as WC ≥ 85 cm in males and ≥ 80 cm in females, as recommended by Guidelines on Preservation and Control Overweight and Obesity in Chinese Adults [8]. Based on new International Diabetes Federation definition, Mets was diagnosed in participants exhibiting central obesity plus at least two of the following criteria: 1) high blood pressure (≥130/85 mmHg and/or taking anti-hypertensive medication), 2) high blood glucose (≥5.6 mmol/L and/or taking anti-diabetic medication), 3) high TG (≥1.7 mmol/L), and 4) low HDL-c (males: < 1.0 mmol/L; females: < 1.3 mmol/L) [9]. Diabetes mellitus (DM) was diagnosed as FBG ≥ 7.0 mmol/L and/or taking anti-diabetic medication. Hypertension was diagnosed as blood pressure ≥ 140/90 mmHg and/or taking anti-hypertensive medication. Drinkers were defined as participants consuming at least 30 g of alcohol per week for at least 1 year [10].

### Arterial stiffness analysis

Arterial stiffness was assessed through automated measurements of cfPWV using a Complior Colson device (Créatech, Besançon, France; for technical characteristics see [11]) in the morning, in a quiet environment, at stable temperature. Participants were assessed in the supine position, after resting for at least 5 min. cfPWV along the artery was measured using two strain gauge transducers (TY-306 pressure-sensitive transducer, Fukuda Denshi Co, Tokyo, Japan), fixed transcutaneously over the course of the arteries, separated by a known distance; the right-side carotid and femoral arteries were used. After waveforms of sufficient quality were recorded, the digitization process was initiated by the operator, and the automatic calculation of the time delay between two upstrokes was obtained. Measurements were repeated over ten cardiac cycles, and the mean value was used in the final analysis. cfPWV was calculated from the measurement of the pulse transit time and distance traveled by the pulse between the two recording sites (measured on the surface of the body, in meters), according to the following formula: cfPWV (m/s) = distance (m)/transit time (s).

### Statistical analysis

Participants were firstly divided into drinkers and non-drinkers, and then subdivided according to the quartiles of serum ALT levels within normal range. In all subgroups, parameters were expressed as mean and standard deviation (in the case of continuous variables with normal distribution), median and interquartile range (in the case of continuous variables with skewed distribution), and number of participants and percentage (in the case of categorical variables). Age, sex, cfPWV, Mets and its components were compared across the subgroups. One-way analysis of variance, Kruskal–Wallis test and χ^2^ test were used to respectively compare differences in continuous variables with normal distribution, continuous variables with skewed distribution and categorical variables. Hazard ratios for prevalence of cfPWV > 10 m/s, Mets and its components with elevated levels of serum ALT were assessed using logistic regression models. Statistical analysis was performed on Statistical Package for Social Sciences (SPSS) version 17.0 (SPSS Inc, Chicago, IL., USA), and statistical significance was defined as a two-tailed *p*-value < 0.05.

## Results

Median (range) age of participants was 53 (18–96) years. Among them, 51.5% were males, and 31.2% were drinkers. Median (range) of serum ALT levels was 16.4 (2.0-40.0) U/L. Prevalence of Mets was 29.4%. Median (range) of cfPWV was 10.1 (2.4-32.7) m/s. In non-drinkers (Table 1), serum ALT levels were significantly related to age, sex, WC, central obesity, hypertension, DM, SBP, DBP, high blood pressure, TG, high TG, HDL-c, FBG, high blood glucose, Mets, cfPWV and cfPWV > 10 m/s (*p* < 0.05 for all). In drinkers (Table 2), serum ALT levels were significantly related to sex, WC, central obesity, SBP, DBP, TG, high TG, HDL-c, low HDL-c, FBG and Mets (*p* < 0.05 for all).

**Table 1:** Characteristics of non-drinkers according to the quartiles of serum ALT levels

Characteristics	Quartile 1 ≤10U/L (*n* = 200)	Quartile 2 11-20U/L (*n* = 871)	Quartile 3 21-30U/L (*n* = 340)	Quartile 4 31-40U/L (*n* = 104)	*P*-value
Age (year)	46(32–63)	60(48–70)	58(50–67)	55(48–63)	<0.001
Males (%)	32(16.0)	269(30.9)	148(43.5)	47(45.2)	<0.001
Central obesity (%)	63(31.5)	525(60.3)	241(70.9)	77(74.0)	<0.001
WC (cm)	75(69–82)	84(77–90)	87(81–94)	87(81–95)	<0.001
Hypertension (%)	43(21.5)	328(37.7)	170(50.0)	46(44.2)	<0.001
DM (%)	13(6.5)	139(16.0)	61(17.9)	19(18.3)	0.002
SBP (mmHg)	115(105–129)	124(112–137)	128(118–141)	126(116–140)	<0.001
DBP (mmHg)	71(66–78)	73(68–80)	79(70–84)	77(70–85)	<0.001
High blood pressure (%)	58(29.0)	427(49.0)	210(61.8)	57(54.8)	<0.001
TG (mmol/L)	0.98(0.77-1.36)	1.35(0.98-1.89)	1.54(1.11-2.20)	1.79(1.18-2.42)	<0.001
High TG (%)	24(12.0)	281(32.3)	149(43.8)	53(51.0)	<0.001
HDL-c (mmol/L)	1.54(1.26-1.79)	1.40(1.20-1.64)	1.30(1.14-1.51)	1.29(1.08-1.52)	<0.001
Low HDL-c (%)	49(24.5)	237(27.2)	107(31.5)	36(34.6)	0.129
FBG (mmol/L)	4.80(4.48-5.06)	4.89(4.50-5.38)	4.95(4.61-5.43)	5.10(4.72-5.55)	<0.001
High blood glucose (%)	21(10.5)	199(22.8)	85(25.0)	29(27.9)	<0.001
Mets (%)	22(11.0)	260(29.9)	149(43.8)	51(49.0)	<0.001
cfPWV (m/s)	9.1(8.0-10.9)	10.3(9.0-12.4)	10.5(9.3-12.3)	10.4(9.0-11.9)	<0.001
cfPWV > 10 m/s	66(33.0)	479(55.0)	213(62.6)	55(52.9)	<0.001

*Abbreviations*: *ALT* alanine aminotransferase, *WC* waist circumference, *DM* diabetes mellitus, *SBP* systolic blood pressure, *DBP* diastolic blood pressure, *TG* triglyceride, *HDL-c* high-density lipoprotein-cholesterol, *FBG* fasting blood glucose, *Mets* metabolic syndrome, *cfPWV* carotid-femoral pulse wave velocity

**Table 2:** Characteristics of drinkers according to the quartiles of serum ALT levels

Characteristics	Quartile 1 ≤10 U/L (*n* = 57)	Quartile 2 11-20 U/L (*n* = 364)	Quartile 3 21-30 U/L (*n* = 187)	Quartile 4 31-40 U/L (*n* = 79)	*P*-value
Age (year)	36(31–57)	43(32–58)	40(31–54)	40(32–54)	0.331
Males (%)	45(78.9)	337(92.6)	180(96.3)	77(97.5)	<0.001
Central obesity (%)	19(33.3)	212(58.2)	133(71.1)	61(77.2)	<0.001
WC (cm)	80(72–87)	86(80–91)	89(84–93)	88(85–95)	<0.001
Hypertension (%)	9(15.8)	101(27.7)	48(25.7)	23(29.1)	0.262
DM (%)	3(5.3)	21(5.8)	10(5.3)	11(13.9)	0.093
SBP (mmHg)	118(111–127)	123(116–135)	125(115–134)	123(117–137)	0.011
DBP (mmHg)	71(67–78)	76(70–81)	77(71–84)	77(71–85)	0.002
High blood pressure (%)	15(26.3)	138(37.9)	77(41.2)	35(44.3)	0.150
TG (mmol/L)	0.93(0.75-1.24)	1.28(0.93-1.74)	1.69(1.14-2.37)	1.74(1.25-2.67)	<0.001
High TG (%)	4(7.0)	99(27.2)	91(48.7)	45(57.0)	<0.001
HDL-c (mmol/L)	1.55(1.36-1.78)	1.35(1.14-1.56)	1.27(1.08-1.48)	1.25(1.01-1.49)	<0.001
Low HDL-c (%)	1(1.8)	44(12.1)	35(18.7)	18(22.8)	0.001
FBG (mmol/L)	4.76(4.40-5.12)	4.85(4.54-5.12)	4.98(4.61-5.27)	4.95(4.52-5.30)	0.030
High blood glucose (%)	5(8.8)	40(11.0)	25(13.4)	17(21.5)	0.060
Mets (%)	3(5.3)	73(20.1)	55(29.4)	35(44.3)	<0.001
cfPWV (m/s)	9.4(8.5-11.4)	9.7(8.7-11.3)	9.5(8.7-10.7)	9.9(8.8-11.2)	0.516
cfPWV > 10 m/s	21(36.8)	166(45.6)	68(36.4)	35(44.3)	0.161

*Abbreviations*: *ALT* alanine aminotransferase, *WC* waist circumference, *DM* diabetes mellitus, *SBP* systolic blood pressure, *DBP* diastolic blood pressure, *TG* triglyceride, *HDL-c* high-density lipoprotein-cholesterol, *FBG* fasting blood glucose, *Mets* metabolic syndrome, *cfPWV* carotid-femoral pulse wave velocity

Hazard ratios for prevalence of Mets, central obesity and high TG significantly increased with elevated levels of serum ALT in both non-drinkers and drinkers (Table 3; *p* < 0.05 for all). Hazard ratios for prevalence of cfPWV > 10 m/s, high blood pressure and high blood glucose significantly increased with elevated levels of serum ALT in non-drinkers (*p* < 0.05 for all), but not in drinkers (*p* ≥ 0.05 for all). Hazard ratios for low HDL-c (*p* < 0.05 for all) significantly increased with elevated levels of serum ALT in drinkers, but not in non-drinkers (*p* ≥ 0.05 for all).

**Table 3:** Hazard ratios for prevalence of cfPWV, Mets and its components with an elevation in serum ALT levels

Serum ALT levels		Non-drinkers		Drinkers	
		HR (95% CI)	*P*-value	HR (95% CI)	*P*-value
Mets	≤10 U/L	1		1	
	11-20 U/L	3.443(2.160-5.487)	<0.001	4.515(1.373-14.851)	<0.001
	21-30 U/L	6.312(3.859-10.324)	<0.001	7.500(2.249-25.011)	<0.001
	31-40 U/L	7.786(4.331-13.996)	<0.001	14.318(4.125-49.699)	<0.001
Central obesity	≤10 U/L	1		1	
	11-20 U/L	3.300(2.377-4.579)	<0.001	2.789(1.548-5.026)	0.001
	21-30 U/L	5.294(3.623-7.734)	<0.001	4.926(2.610-9.296)	<0.001
	31-40 U/L	6.202(3.649-10.539)	<0.001	6.778(3.165-14.512)	<0.001
High TG	≤10 U/L	1		1	
	11-20 U/L	3.493(2.228-5.475)	<0.001	4.950(1.746-14.035)	0.003
	21-30 U/L	5.721(3.550-9.220)	<0.001	12.560(4.369-36.107)	<0.001
	31-40 U/L	7.621(4.292-13.532)	<0.001	17.537(5.782-53.191)	<0.001
Low HDL-c	≤10 U/L	1		1	
	11-20 U/L	1.152(0.808-1.643)	0.435	7.700(1.040-57.029)	0.046
	21-30 U/L	1.415(0.953-2.101)	0.085	12.895(1.726-96.357)	0.013
	31-40 U/L	1.631(0.973-2.735)	0.063	16.525(2.136-127.859)	0.007
High blood pressure	≤10 U/L	1		1	
	11-20 U/L	2.355(1.688-3.285)	<0.001	1.710(0.914-3.199)	0.093
	21-30 U/L	2.969(1.815-4.858)	<0.001	1.960(1.016-3.783)	0.045
	31-40 U/L	3.955(2.716-5.758)	<0.001	2.227(1.065-4.659)	0.033
High blood glucose	≤10 U/L	1		1	
	11-20 U/L	2.524(1.564-4.075)	<0.001	1.284(0.484-3.403)	0.615
	21-30 U/L	2.841(1.699-4.753)	<0.001	1.605(0.585-4.405)	0.358
	31-40 U/L	3.296(1.768-6.145)	<0.001	2.852(0.985-8.256)	0.053
cfPWV > 10 m/s	≤10 U/L	1		1	
	11-20 U/L	2.481(1.795-3.429)	<0.001	1.437(0.808-2.558)	0.217
	21-30 U/L	3.405(2.358-4.918)	<0.001	0.980(0.530-1.812)	0.948
	31-40 U/L	2.279(1.403-3.701)	0.001	1.364(0.679-2.740)	0.384

*Abbreviations*: *cfPWV* carotid-femoral pulse wave velocity, *Mets* metabolic syndrome, *ALT* alanine aminotransferase, *HR* hazard ratio, *CI* confidential interval, *TG* triglyceride, *HDL-c* high-density lipoprotein-cholesterol

## Discussion

Prevalence of Mets is rapidly increasing in parallel with an elevation in serum ALT levels [1]. Serum ALT levels are widely used to monitor liver function, and patients with Mets often display elevated levels of serum ALT [12, 13]. Previous studies have suggested that Mets can be viewed as a strong risk factor for an elevation in serum ALT levels [3–6]. However, few community-based studies have addressed the relationship between Mets and serum ALT levels within normal range in a Chinese community-dwelling population. In both non-drinkers and drinkers, the current analysis revealed that prevalence of Mets increased with an elevation in serum ALT levels. In both preclinical population and clinical patients, insulin resistance is an essential condition for the accumulation of liver fat and plays a key role in the development of Mets [14, 15]. Giving the relationship between Mets and serum ALT levels suggested by the current analysis, an elevation in serum ALT levels should receive more attention in clinical practice, as a factor not only reflecting change of liver fat, but also relating to insulin resistance [1].

Regarding the components of Mets, the current analysis showed that central obesity and high TG become more common as serum ALT levels increased, regardless of alcohol intake [16]. Several studies have demonstrated that change in lipid metabolism has important effects on liver insulin resistance and serum ALT levels [17]. Other studies have also suggested that weight control is as important as improving lipid metabolism on controlling insulin resistance and normalizing serum ALT levels [18–21]. Compared with adipose tissue in other body parts, visceral adipose tissue is more resistant to insulin, and hyperinsulinemia promotes the liver lipogenesis and raises the serum ALT levels [22, 23]. As for other components of Mets, the current analysis showed that prevalence of high blood pressure and glucose increased with an elevation in serum ALT levels in non-drinkers, but not in drinkers. In other words, the relationship of serum ALT levels to high blood pressure and glucose was dissimilar in participants with and without alcohol intake. The current analysis confirmed the empirical knowledge in clinical practice, and demonstrated that alcohol intake can increase the enzyme activity of ALT in the liver, with important effects on the relationship between different components of Mets and serum ALT levels. However, further studies are needed to clarify the potential relationship of serum ALT levels to high blood pressure and glucose.

To our knowledge, few community-based studies have attempted to determine the possible relationship between cfPWV and serum ALT levels within normal range across the world. Therefore, another important finding of the current analysis was the relationship between cfPWV and serum ALT levels even within normal range of serum ALT levels in non-drinkers, but not in drinkers. According to the current analysis, reasons for the relationship between cfPWV and serum ALT levels are related to the intermediary role of insulin resistance and Mets. The overlooked relationship between cardiovascular and liver diseases should receive more attention by clinical doctors. Moreover, the current analysis stressed that arterial stiffness is a breakthrough point in exploring the relationship between cardiovascular and liver diseases in both scientific research and clinical practice.

## Conclusions

In a Chinese community-dwelling population, the current analysis revealed that prevalence of Mets and its components (including central obesity and high TG) increased with an elevation in serum ALT levels within normal range in both non-drinkers and drinkers, while cfPWV and other components of Mets, such as high blood pressure and glucose, increased with an elevation in serum ALT levels in non-drinkers, but not in drinkers.

## Abbreviations

ALT: Alanine aminotransferase
cfPWV: Carotid-femoral pulse wave velocity
DBP: Diastolic blood pressure
DM: Diabetes mellitus
FBG: Fasting blood glucose
HDL-c: High-density lipoprotein-cholesterol
Mets: Metabolic syndrome
SBP: Systolic blood pressure
TG: Triglyceride
WC: Waist circumference
